# Temperature Shapes Ecological Dynamics in Mixed Culture Fermentations Driven by Two Species of the *Saccharomyces* Genus

**DOI:** 10.3389/fbioe.2020.00915

**Published:** 2020-08-21

**Authors:** Eva Balsa-Canto, Javier Alonso-del-Real, Amparo Querol

**Affiliations:** ^1^(Bio)process Engineering Group, IIM-CSIC, Vigo, Spain; ^2^Grupo de Biología de Sistemas en Levaduras de Interés Biotecnológico, IATA-CSIC, Valencia, Spain

**Keywords:** mixed culture fermentations, *Saccharomyces*, ecological modeling, Lotka-Volterra, Gilpin-Ayala

## Abstract

Mixed culture wine fermentations combining species within the *Saccharomyces* genus have the potential to produce new market tailored wines. They may also contribute to alleviating the effects of climate change in winemaking. Species, such as *S. kudriavzevii*, show good fermentative properties at low temperatures and produce wines with lower alcohol content, higher glycerol amounts and good aroma. However, the design of mixed culture fermentations combining *S. cerevisiae* and *S. kudriavzevii* species requires investigating their ecological interactions under cold temperature regimes. Here, we derived the first ecological model to predict individual and mixed yeast dynamics in cold fermentations. The optimal model combines the Gilpin-Ayala modification to the Lotka-Volterra competitive model with saturable competition and secondary models that account for the role of temperature. The nullcline analysis of the proposed model revealed how temperature shapes ecological dynamics in mixed co-inoculated cold fermentations. For this particular medium and species, successful mixed cultures can be achieved only at specific temperature ranges or by sequential inoculation. The proposed ecological model can be calibrated for different species and provide valuable insights into the functioning of alternative mixed wine fermentations.

## 1. Introduction

Wine fermentation is a complicated ecological and biochemical process in which a wide variety of yeasts produce ethanol and contribute to the sensory attributes of the final product. Still, the alcohol-tolerant strains of *Saccharomyces cerevisiae* ever dominate the late stages of spontaneous fermentations (Bauer and Pretorius, 2000; Cray et al., 2013). *S. cerevisiae* strains show strong competitive fitness when sharing the fermentative environments with other *Saccharomyces* and *non-Saccharomyces* yeasts, in part thanks to its ability to consume sugars in the presence of oxygen (Crabtree effect). This property offers a competitive advantage over other *non-cerevisiae* or *non-Saccharomyces* species which are displaced sooner or later during the process (Holm et al., 2001; Arroyo-López et al., 2011; Alonso-del-Real et al., 2017).

The output of spontaneous fermentations depends on the amounts and variety of yeasts and bacteria present in the must, grape variety and maturity and process conditions (e.g., temperature, aeration, etc.). However, the variability, mostly in the must flora and grape chemical properties, results in severe difficulties to predict and reproduce wine properties. The use of starters has dramatically improved the reliability of the process and the quality of wines (Pretorious, 2000).

*S. cerevisiae* yeast starters are universally preferred to initiate fermentation processes. However, the new challenges faced by the wine industry such as those related to climate change, or the consumers' demands for lower-alcohol wines, call for the use of alternative starting strategies (Goold et al., 2017; Querol et al., 2018).

Recent evidence suggests that mixed culture processes combining *S. cerevisiae* with non-conventional *Saccharomyces* species have the potential to produce new market tailored wines (Fleet, 2008; Padilla et al., 2017). Besides, cryotolerant non-*cerevisiae* species may contribute to alleviating the effects of climate change in winemaking (Pérez-Torrado et al., 2018). Species such as *S. uvarum* or *S. kudriavzevii* show good fermentative properties at low temperatures and produce wines with lower alcohol content, higher glycerol amounts and good aromatic profiles (Gangl et al., 2009; Gamero et al., 2013; Alonso-del-Real et al., 2017). What remains unclear is what are the fermentation conditions, i.e., temperature and initial inoculum, so that *S. cerevisiae* does not exclude other species in mixed co-inoculated cultures.

The primary aim of this work is to address this question through a model-based approach for the particular case of mixed culture fermentations involving *S. cerevisiae* and *S. kudriavzevii* strains. The underlying idea is to obtain an ecological model whose parameters vary with the temperature to recover the dynamics of both cells' populations under cold to mild temperature regimes.

In ecology, the classical Lotka-Volterra model (LV, Volterra, 1926; Lotka, 1932) considers that the various species compete for resources, and sums up the role of intraspecific -within the same species- and interspecific - between different species- competitive effects. Both effects are considered to depend linearly on the cellular density. The LV model has been largely used to model microbial interactions in various environments (Mounier et al., 2008; Stein et al., 2013; Berry and Widder, 2014; Cadavez et al., 2019).

Alternatives to the LV model have become increasingly abundant in the literature (see, for example, the recent work by Gavina et al., 2018 and the works cited therein). Generalized versions of the LV model allow for more flexibility by including non-linear intraspecific and interspecific competition terms. The underlying idea is to account for higher-order interactions. Gilpin and Ayala (1973) proposed a non-linear intraspecific competition, and others added non-linear decay terms (see the recent work by Gavina et al., 2018 for various examples). Frequency-dependent or saturable competition alternatives account for the aggregated effects of the production of toxic compounds, crowding or cross-feeding (MacLean and Gudelj, 2006; Gore and van Oudenaarden, 2009; Ribeck and Lenski, 2015).

Lately, we have shown that measuring mixed microbial density, even if the number of sampling times is significant, is not enough to fully identify models that predict the ecology of mixed co-inoculated cultures (Balsa-Canto et al., 2020). Therefore, in this work, we combine individual growth time-series data in single and mixed cultures obtained at cold temperatures and a systematic model building approach to defining the ecological model that offers the best compromise between complexity, goodness-of-fit and cross-validation. The derived model is the first, to the authors' knowledge, to simultaneously recover single and mixed yeast dynamics in cold fermentations. Its nullclines offer valuable information concerning the role of temperature in the coexistence of both species.

## 2. Materials and Methods

### 2.1. Multi-Experiment Design

#### 2.1.1. Synthetic Must Fermentations

We chose a commercial strain, T73 (Lalvin T73 from Lallemand Montreal, Canada), as our wine *S. cerevisiae* representative and *S. kudriavzevii* strain CR85, a natural isolate from oak tree bark in Agudo, Ciudad Real, Spain. From now on, we regard strains as *Sc* and *Sk*, respectively.

Fermentations were performed in 3*x* or 6*x* replicates in 250 mL flasks that contained 200 mL of synthetic must (SM), which is frequently used in microvinification experiments (Rossignol et al., 2003), with 100 g/L of glucose and 100 g/L of fructose.

To assess the relative growth of *S. cerevisiae* and *S. kudriavzevii* species under winemaking conditions, we performed mixed culture experiments in which we measured the relative amount of both strains. As controls, we monitored the growth of each strain in mono-cultures under the same conditions as the mixed culture experiments. Overnight precultures were grown in yeast extract–peptone–dextrose (YPD) medium at 25°C. Afterwards must was inoculated with the corresponding yeast strain to reach an initial concentration of 10^6^ cells/mL, and was incubated at a fixed temperature (8, 12, 20, or 25°C) with agitation at 100 rpm during fermentation.

Cell samples were collected at several time points during fermentation and kept at −20°C for the subsequent total DNA isolation, used for the quantitative polymerase chain reaction (qPCR) analyses, as described below. Cell counting was carried out in a Neubauer chamber to determine cell density at every sampling point. Growth curves were obtained by considering cell density and the proportion of competing strains given by the qPCR data.

Müller valves were used to monitor fermentation stage through weight loss until it reached a constant weight when it was considered to be over.

#### 2.1.2. qPCR Assays

To obtain the relative concentration of both strains in each biological replicate, we followed a qPCR based strategy as detailed in Alonso-del-Real et al. (2017). We designed species-specific primers for *S. cerevisiae* and *S. kudriavzevii* on a region corresponding to a fragment of the gene *BUD3* for relative quantification of both yeasts.

### 2.2. Ecological Primary Models

**Figure 1:**
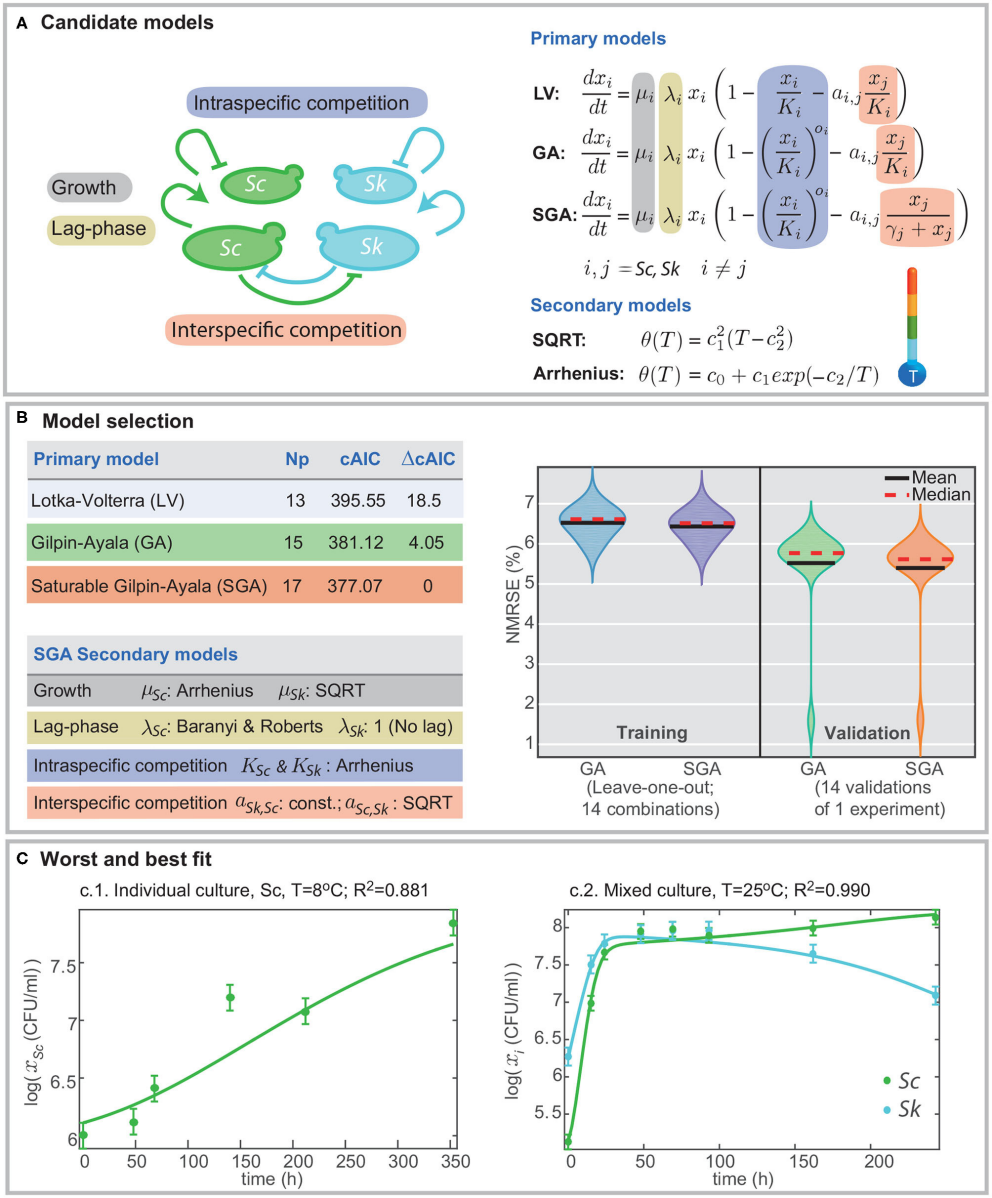
Formulation of candidate models and model selection. **(A)** presents the different mechanisms included in the models, namely, growth, lag-phase, intra- and interspecific competition. Different definitions for intra- and interspecific competition result in different candidate models: Lotka-Volterra (LV), Gilpin-Ayala (GA), and Gilpin-Ayala with saturable competition (SGA). **(B)** presents the comparison of the best-fitted models. SGA is the best model; its fit to the multi-experiment data set results in better cAIC and the minimum training and validation errors in cross-validation. **(C)** shows the worst and best fit to the data for the multi-experiment model calibration.

We defined three primary models as candidates to describe the ecology of the mixed cultures, i.e., to predict the individual density in a mixed culture. The classical Lotka-Volterra model underlies all candidate models (see Figure 1 for exact formulations). Lotka (1932) and Volterra (1926) proposed a widely known and used model to describe population dynamics of species competing for common resources (e.g., nutrients) (Murray, 2001). The model accounts for intraspecific and interspecific competition. To do so, the model assumes logistic growth of the species and considers that interspecific interactions are linear, i.e., that a fraction of the competing species contributes linearly to reduce growth rate. Gilpin and Ayala (1973) found out that the classical Lotka-Volterra model could not describe those cases in which intraspecific competence, or growth regulation, is non-linear. Later, Thébault and Fontaine (2010) showed that the influence of one species on another might lag in time or saturate and proposed a non-linear interspecific interaction.

All these models can be embedded in the following mathematical formulation:

(1) $$\begin{array}{l} \frac{dx_{i}}{dt}=G\left(x_{i},x_{j}\right)\\ \;=\mu_{i}x_{i}\left(1-{\left(\frac{x_{i}}{K_{i}}\right)}^{\theta_{i}}-a_{i,j}f_{c}\left(x_{j}\right)\right)\;i,j=Sc,Sk\;i\neq j \end{array}$$

where *i* can be either *Saccharomyces cerevisiae* (*Sc*) or *Saccharomyces kudriavzevii* (*Sk*) and *j* will be either *Sk* or *Sc* with *i* ≠ *j*; *x*_*i*_ corresponds to the cell density for both species; μ_*i*_ corresponds to the specific growth rate; *K*_*i*_ is the so-called carrying capacity for species *i*; θ_*i*_ ≥ 1 controls the degree of non-linearity in intraspecific growth regulation. Coefficients *a*_*i,j*_ measure the competitive strength of species *j* on *i*. If *a*_*i,j*_ > 0 species or strains are said to be competing. *f*_*c*_(*x*_*j*_) describes the interspecific competition, which depends linearly on *x*_*j*_ for the case of the Lotka-Voterra (LV) and Gilpin-Ayala (GA) models while Holling type II function is used to describe saturable or delayed interactions (SGA). Note that if *a*_*i,j*_ = 0 and *a*_*j,i*_ = 0, Equation (1) are transformed into the Richards model for single cultures.

### 2.3. Lag-Phase Description

Previous models can be modified to include the role of a certain inhibition before the exponential phase, i.e., the so called lag-phase, *l*_*i*_(*T*) which may, in principle, vary with the environment. Equations (1) would read:

(2) $$\begin{array}{l} \frac{dx_{i}}{dt}=l_{i}\left(T\right)G\left(x_{i},x_{j}\right) \end{array}$$

Following Baranyi and Roberts (1994) we defined the lag-phase as follows:

(3) $$\begin{array}{ll} l_{i}=\frac{l_{i,0}\left(T\right)}{l_{i,0}\left(T\right)+\left(1-l_{i,0}\left(T\right)e^{\left(-\mu_{i}\left(T\right)t\right)}\right)} & 0\leq l_{i}\left(T\right)\leq 1 \end{array}$$

### 2.4. Secondary Models

Primary models (Equation 1) determine the magnitude of the responses of interest, such as maximum specific growth rate, lag phase or the time to reach a specified level. Nevertheless, μ_*i*_, *K*_*i*_ and *a*_*i,j*_ parameters may depend on the environment, i.e., the temperature in this work. Secondary models describe this dependence (McMeekin et al., 2002).

Taking into account previous data on the dependency of μ with the temperature (Salvadó et al., 2011), we proposed two secondary models with a maximum of three parameters each: the Ratkowsky or squared-root model (SQRT) Ratkowsky et al. (1983) and the well-known Arrhenius equation (A):

(4) $$\begin{array}{l} \mu_{SQRT}\;:\;\mu_{i}\left(T\right)=\mu_{i,0}^{2}{\left(T-\mu_{i,1}\right)}^{2} \end{array}$$

(5) $$\begin{array}{l} \mu_{A}\;:\;\mu_{i}\left(T\right)=\mu_{i,0}+\mu_{i,1}e^{\left(-\frac{\mu_{i,2}}{T}\right)} \end{array}$$

Similarly for the carrying capacity and the competition parameters we define:

(6) $$\begin{array}{l} K_{i,SQRT}\;:\;K_{i}\left(T\right)=K_{i,0}^{2}{\left(T-K_{i,1}\right)}^{2} \end{array}$$

(7) $$\begin{array}{l} K_{i,A}\;:\;K_{i}\left(T\right)=K_{i,0}+K_{i,1}e^{\left(-\frac{K_{i,2}}{T}\right)} \end{array}$$

and:

(8) $$\begin{array}{l} a_{i,j,SQRT}\;:\;a_{i,j}\left(T\right)=a_{i,j,0}^{2}{\left(T-a_{i,j,1}\right)}^{2} \end{array}$$

(9) $$\begin{array}{l} ai,j,A\;:\;a_{i,j}\left(T\right)=a_{i,j,0}+a_{i,j,1}e^{\left(-\frac{a_{i,j,2}}{T}\right)} \end{array}$$

Note that the Arrhenius equations can also explain the case in which the biological parameter is constant with the temperature (with *a*_*i,j*,1_ = 0).

Note that subscripts 0, 1, and 2 identify the parameters required to define the secondary models. Two parameters with subscripts 0 and 1 are required for the Ratkowski temperature dependency while three subscripts 0, 1, and 2 identify the three parameters required to define the Arrhenius temperature dependency.

### 2.5. Inferring Ecological Interactions and the Role of the Temperature in One Step

We used data fitting to infer the best combination of primary and secondary models. We considered three cases, each corresponding to one of the primary models, namely LV, GA, and SGA. At the same time, we defined a mixed integer-real formulation to select the best secondary model automatically. For that purpose, we redefined the biological parameters as follows:

(10) $$\begin{array}{ll} \mu_{i}\left(T\right)=w_{\mu ,i}\mu_{SQRT,i}+\left(1-w_{\mu ,i}\right)\mu_{A,i} & i=Sc,Sk \end{array}$$

(11) $$\begin{array}{ll} K_{i}\left(T\right)=w_{K,i}K_{SQRT,i}+\left(1-w_{K,i}\right)K_{A,i} & i=Sc,Sk \end{array}$$

(12) $$\begin{array}{ll} a_{i,j}\left(T\right)=w_{a,i,j}a_{SQRT,i,j}+\left(1-w_{a,i,j}\right)a_{A,i,j} & i,j=Sc,Sk \end{array}$$

in such a way that *w*_μ,*i*_, *w*_*K,i*_, and *w*_*a,i,j*_ are binary parameters which take the value 1 for *SQRT* and 0 for Arrhenius temperature dependence. Data fitting aims at computing the best combination of binary and real parameters, providing the best secondary model plus the corresponding parameters.

The optimal parameters for the candidate models correspond to those that maximize the likelihood of the data, e.g., those that minimize the following log-likelihood function:

(13) $$\begin{array}{l} L=\overset{n_{exp}}{\underset{k=1}{\sum }}\overset{n_{obs}}{\underset{j=1}{\sum }}\overset{n_{st}}{\underset{i=1}{\sum }}{\left(\frac{x_{k,j,i}^{m}-x_{k,j,i}^{d}}{\sigma_{k,j,i}}\right)}^{2}, \end{array}$$

where *n*_*exp*_ corresponds to the number of experiments (single and mixed cultures); *n*_*obs*_ corresponds to the number of observed quantities, i.e., *x*_*Sc*_&*x*_*Sk*_ in single and mixed culture experiments and *n*_*st*_ corresponds to the number of sampling times per experiment. *x*^*m*^ regards the model predictions and *x*^*d*^ the measured data. $\sigma_{k,j,i}^{2}$ regards the data noise variance as computed from the experimental replicates.

We selected the best model attending to the Akaike's information criterion (AIC) and the quality in cross-validation. The AIC intends to balance parsimony and relative information loss across candidate models, penalizing the number of parameters to avoid over-fitting, its value for each candidate model *M* reads:

(14) $$\begin{array}{l} AIC_{M}=L\left({\text{p}}^{*}\right)+2n_{p}+2n_{p}\left(n_{p}+1\right)/\left(n_{d}-n_{p}-1\right) \end{array}$$

being p^*^ the optimum value of model parameters as found in parameter estimation; *n*_*p*_ the number of parameters in the model *M* and *n*_*d*_ the number of data. The minimum AIC value (AIC_*min*_) was used to re-scale the Akaike's information criterion. The re-scaled value Δ_*M*_ = AIC_*M*_-AIC_*min*_ was used to assess the relative merit of the model: models such as Δ_*M*_ ≤ 2 have substantial support, models for which 4 ≤ Δ_*M*_ ≤ 7 have considerably less support and models with Δ_*M*_ > 10 have no support Burnham and Anderson (2004).

We also compared models by cross-validation. For each trial, we split the data into two datasets—the “training” dataset and the “validation” dataset. We defined the “training” dataset by leaving one experiment out at each iteration (the “validation” experiment). In this way, we solved the data fitting problem using 13 experiments and used the optimal parameters to assess the predictive abilities of the model with the “validation” experiment. We ran all trials for all candidate models and computed the normalized mean squared error for training and validation. The best model was the one reporting the best compromise between training and validation over all trials.

### 2.6. Numerical Methods

Data fitting and model selection problems were solved using the numerical approaches available in AMIGO2 toolbox (Balsa-Canto et al., 2016). Specifically, CVODES (Hindmarsh et al., 2005) was used to solve the model while the enhanced Scatter Search method (eSS, Egea et al., 2009) was used to optimize parameters.

## 3. Results

### 3.1. A Gilpin-Ayala Model With Saturable Competition Best Describes Mixed Culture Dynamics

Data-fitting results reveal that the Gilpin-Ayala model with and without saturable competition perform better than the Lotka-Volterra model. Figure 1B summarizes the statistics for the three primary models showing a significant difference in the goodness-of-fit between the LV and the GA models (Δ*cAIC* > 18), thus signifying the critical role of the non-linear intraspecific competence.

The minimum AICc value corresponds to the SGA model. Still, the re-scaled AIC for the GA model is around 4; thus indicating that SGA and GA models offer in practice the same quality. Besides, both models perform well at cross-validation, with validation errors around the 6% (Figure 1B). Nevertheless, the SGA outcompetes the GA model for all cross-validation trials but for the one in which we omit the single culture of *S. cerevisiae* at *T* = 25°C.

Figure 1C presents the worst and the bests fits. The quality of fits is visually good; the *R*^2^ values range from 0.881 for the worst case, corresponding to the single culture of *Sc* at *T* = 8°C, and 0.990 for the best case, corresponding to a mixed culture at *T* = 25°C. The *R*^2^ value for the simultaneous fitting of all experiments, single and mixed, is 0.973.

Table 1 presents the optimal parameter values for the SGA model plus their associated uncertainty as computed through cross-validation. As can be seen from the table, the maximum relative uncertainty is around 16.0%. Figure S1 shows the uncertainty associated with the predictions.

**Table 1:** Best model parameter values and associated uncertainty.

**Species**	**Biological**	**Secondary**	**Model**	**Optimal**	**% Uncertainty**
	**parameter**	**model**	**parameter**	**value**	
*S. cerevisiae*	μ_*Sc*_(*T*)	*Arrhenius*	μ_*Sc*,0_	1.39 × 10^−2^	15.2
			μ_*Sc*,1_	3.68	7.03
			μ_*Sc*,2_	5.54 × 10^1^	2.30
	*K*_*Sc*_(*T*)	*Arrhenius*	*K*_*Sc*,0_	8.02	0.11
			*K*_*Sc*,1_	1.70 × 10^1^	13.4
			*K*_*Sc*,2_	1.08 × 10^2^	3.92
	*l*_*Sc*_	-	*l*_*Sc*,0_	3.64 × 10^−1^	16.4
	θ_*Sc*_	-	θ_*Sc*_	5.55 × 10^−1^	7.59
	*a*_*Sc,Sk*_	*SQRT*	*a*_*Sc,Sk*,0_	4.63 × 10^−3^	13.1
			*a*_*Sc,Sk*,1_	0.00	0.00
	γ_*Sk*_	-	γ_*Sk*_	8.63	0.84
*S. kudriavzevii*	μ_*Sk*_(*T*)	*SQRT*	μ_*Sc*,0_	1.89 × 10^−2^	1.62
			μ_*Sc*,1_	0.00	0.00
	*K*_*Sk*_(*T*)	*Arrhenius*	*K*_*Sk*,0_	7.94	0.2
			*K*_*Sk*,1_	4.96 × 10^1^	5.38
			*K*_*Sk*,2_	1.32 × 10^2^	1.65
	*l*_*Sk*_	-	*l*_*Sk*,0_	1.00	0.00
	θ_*Sk*_	-	θ_*Sk*_	7.07 × 10^−1^	8.38
	*a*_*Sk,Sc*_	*cte*	*a*_*Sk,Sc*,0_	5.99	4.27
	γ_*Sc*_	-	γ_*Sc*_	8.92	0.27

*The table reports the optimal parameter values for the best model plus their associated uncertainty as computed through cross-validation. For those biological parameters that depend on the temperature, the best secondary model is reported*.

### 3.2. Temperature Modulates the Specific Growth Rate, the Maximum Yield, and the Lag-Phase of the Individual Cells

**Figure 2:**
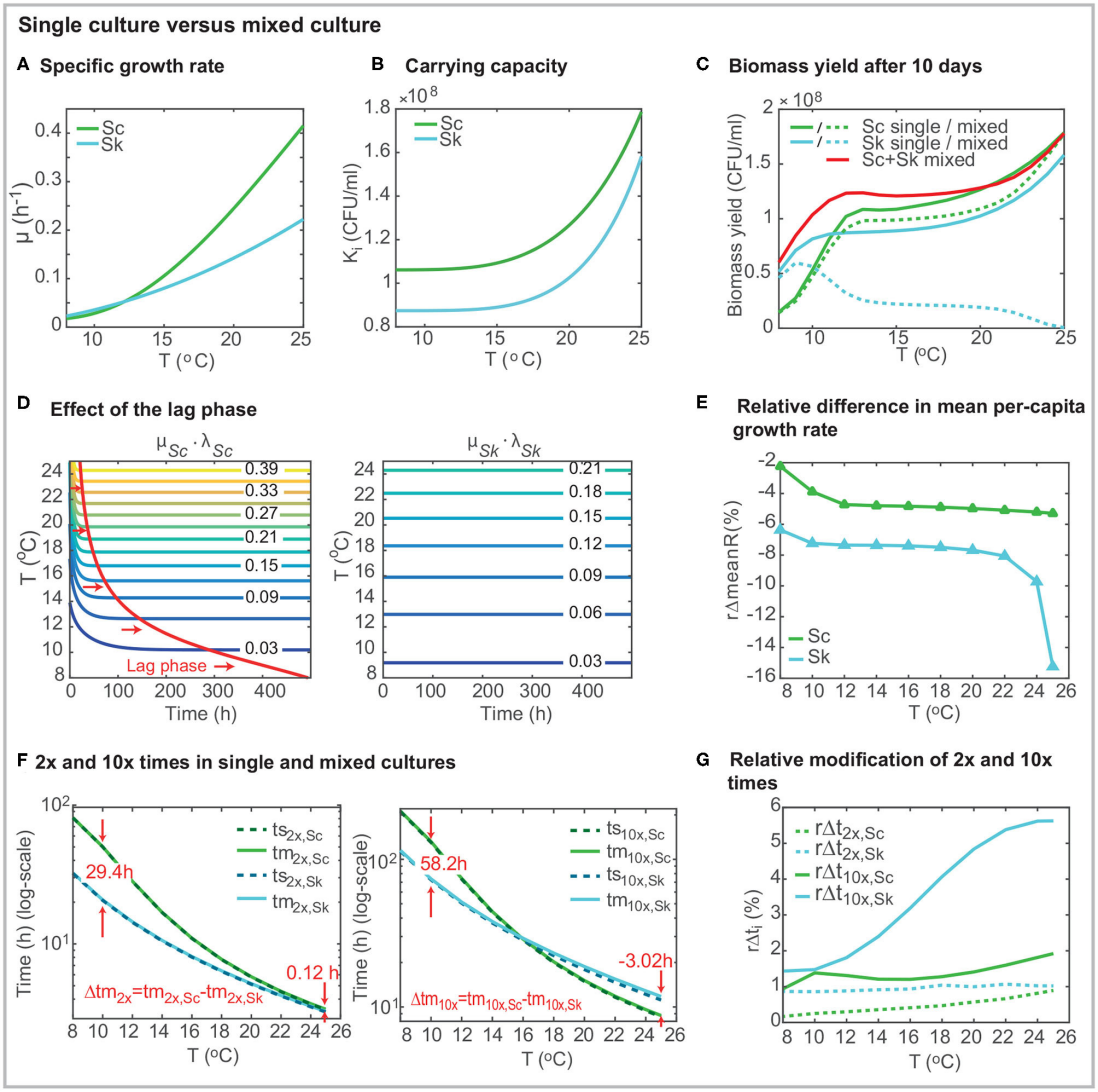
Comparison of strain behavior in single and mixed cultures using the best model. **(A)** presents the specific growth rate μ_*i*_(*T*) as a function of the temperature for both strains. **(B)** shows the carrying capacity as a function of the temperature (*K*_*i*_(*T*)). **(C)** shows the biomass yield after 10 days in mixed co-inoculated (50/50) fermentations. **(D)** shows the effect of the lag-phase. In the case of *S. cerevisiae*, the time required to achieve the maximum specific growth rate depends on the temperature. The lag-phase is significantly longer at lower temperatures. **(E)** presents the relative decrease of the mean per-capita growth rate (over time) in mixed culture. The per-capita growth rate for *Sk* in mixed cultures (*R*_*m,Sk*_) is up to a 15.4% lower than its value in single culture (*R*_*s,Sk*_). For the case of *Sc*, the effect of temperature on the per-capita growth rate is higher than the presence of another cell. **(F)** presents the 2x and 10x times for single and mixed cultures. **(G)** shows the relative increase in 2*x* and 10*x* times in mixed culture. The presence of *Sc* has a significant effect on the 10*x* time for *Sk*. Figures were obtained for a 2 × 10^6^(*CFU*/*ml*), 50% of each species (50/50).

The specific growth rate increases with the temperature for both species (see Figure 2A) and follows the Arrhenius equation for *Sc* and SQRT secondary model for *Sk*. Figure 2A presents a comparison of the specific growth rate for both species showing how only at low temperatures (≤ 12°C) the value for *Sk* is higher than that for *Sc*. From Figure 2A, it is apparent that the temperature has a significant effect on the specific growth rate, particularly for *Sc*. For the case of *Sk* the specific growth rates increases from 0.023 to 0.222 (around 10 times) while for *Sc*, it increases from 0.018 to 0.415 within the range of temperatures (more than 20 times).

The carrying capacities, *K*_*Sc*_ &*K*_*Sk*_, which coincide with the final cell counts in single cultures, increase with the temperature following an Arrhenius dependency for both species (Table 1, Figure 2B). The carrying capacity for *Sc* is above the carrying capacity for *Sk* independently of the temperature. Interestingly *K*_*Sk*_ increases more rapidly with the temperature than *K*_*Sc*_ (*K*_*Sc*_ increases a 68% from 8 to 25°C while *K*_*Sk*_ increases an 81%) while the opposite behavior was observed with the specific growth rates.

Figure 2C presents the biomass yield for single and mixed co-inoculated (50/50) cultures after 10 days (standard duration of a cold mixed fermentation). What stands out in the figure is that the total biomass yield is higher in mixed cultures than in single cultures for temperatures lower than around 20°C. The final amount of *Sk* decreases with the temperature. On the contrary, the final amount of *Sc* increases with temperature. Its value is, in general, being lower than in single culture but at high temperatures in which *x*_*Sc*_(*t*_*f*_) ≈ *K*_*Sc*_. The yield of *Sc* in mixed cultures, is higher than the yield of *Sk*, for *T* > 10°C.

It should be noted that cold temperatures induce a lag phase in *S. cerevisiae* (see Figure 2D). Lag parameters (*l*_*Sc*,0_ and *l*_*Sk*,0_) were considered for both strains during the parameter estimation. For all runs, the parameter for *Sk* converged to its maximum value 1. Thus no lag effect is obtained for *Sk*. However, the lag phase parameter value for *Sc* is 0.364 (see Table 1) inducing a delay in growth which is being reduced as the temperature increases. Figure 2D shows this effect: the specific growth rate is only achieved after around 490*h* at very low temperatures while it is rapidly achieved (around 21 *h*) at *T* = 25°C. Despite the lag phase, *Sc* maximum growth rate (*max*(μ_*i*_·*l*_*i*_(*t*))) is higher than the maximum *Sk* growth rate for *T* >= 12.16°C in single cultures.

### 3.3. *S. cerevisiae* Accelerates Its Growth in the Presence of *S. kudriavzevii*

The per-capita growth rate is defined as $R\left(t\right)=\frac{1}{x_{i}}\frac{dx_{i}}{dt}$. We computed its value for each strain in single (*R*_*s*_(*t*)) and mixed culture (*R*_*m*_(*t*)). Figures S2A–D present *R*_*s*_(*t*) and *R*_*m*_(*t*) for various temperature values. *Sc* per-capita growth rate increases till a maximum value before decreasing to 0 which would correspond to the stationary phase (*x*_*Sc*_ = *K*_*Sc*_, in single cultures; *max*(*X*_*Sc*_) in mixed cultures). *Sk* per-capita growth rate starts at its maximum at *t* = 0 and decays till the end of the process for both single and mixed cultures. Note that in mixed cultures, the per-capita growth rate becomes negative, indicating that the population decays—the more negative the most important the decay. Indeed, the simulation of the system dynamics reveals an overshoot of *Sk* population followed by a collapse till the final *Sk* value is achieved (data shown in Figure S4).

The comparison of the per-capita growth rate in single and mixed cultures (*R*_*m*_(*t*) − *R*_*s*_(*t*), Figures S2E,F) reveals that *Sk* is slower in mixed cultures throughout the process and independently of the temperature (*R*_*m,Sk*_(*t*) − *R*_*s,Sk*_(*t*) ≤ 0 in *t* ∈ [0, *t*_*f*_]). Note that this difference is ≤ 1% at the beginning of the culture for all temperatures, indicating that the maximum per-capita growth rate is not significantly affected by the presence of another cell at early times. However, at higher temperatures, the per-capita growth rate in mixed cultures is significantly lower than in single cultures, this would correspond to the decay (exclusion) of *Sk* (see Figure S2).

On the contrary, at some point during the process, *Sc* per-capita growth rate in mixed cultures is higher than in single cultures (*R*_*m,Sc*_(*t*) − *R*_*s,Sc*_(*t*) > 0). For example, at *T* = 25°C the per-capita growth rate from 28.3 *h* on, is higher for *Sc* in the presence of *Sk*. The lower the temperature, the lower the effect, but still this “acceleration” is observed at all tested temperatures. It is also relevant that the growth rate difference is less than 7% for all cases.

Figure 2E presents the relative difference in the mean over-time per-capita growth rate as a function of temperature ($r\Delta meanR_{i}=100\times \frac{mean\left(R_{m,i}\left(t\right)\right)-mean\left(R_{s,i}\left(t\right)\right)}{max\left(R_{s,i}\right)}$ with *t* ∈ [0, 10 *days*]). The figure emphasizes that the impact of the presence of *Sc* over *Sk* mean per-capita growth rate is higher than the impact of *Sk* over *Sc*. *Sk* mean per-capita growth rate decreases substantially: between a 6.4% at *T* = 8°C a 15.2% at *T* = 25°C; while *Sc* mean per-capita growth rate decreases less than a 5.4% within the temperature range.

We also used the model to compute the (first) population doubling 2*x* time and 10*x* time for each species as a function of the temperature in both single (*ts*_2*x,i*_ and *ts*_10*x,i*_) and mixed cultures (*tm*_2*x,i*_ and *tm*_10*x,i*_). The doubling time regards the time required to achieve *X*_*i*_ = 2 × *X*_*i*,0_ and the 10*x* time regards the time required to achieve *X*_*i*_ = 10 × *X*_*i*,0_. Remark that their values are affected by the lag-phase and the presence of a second species in mixed cultures. Results for $X_{i,0}=1\times 10^{6}$ CFU/ml are shown in Figure 2F.

**Figure 3:**
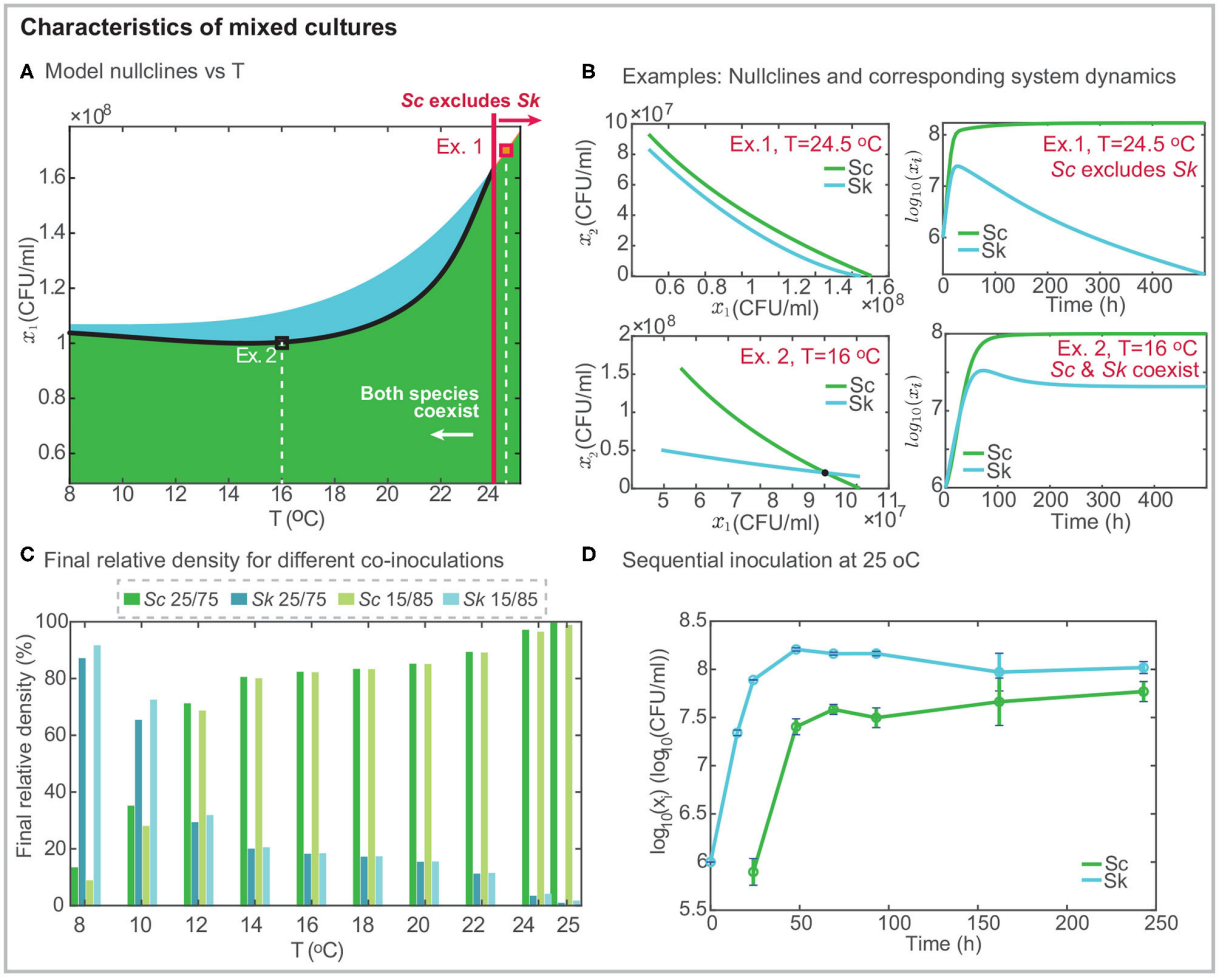
Characteristics of mixed cultures. **(A)** presents the projection of the model nullclines over the temperature and the final density of *Sk*; the black line shows the intersection between the nullclines indicating the range of temperatures for which both cells coexist independently of the initial inoculation. Black and red squares mark two different examples: Ex. 1 at *T* = 24.5°C in which *Sc* excludes *Sk* and Ex.2 at *T* = 16°C in which both strains coexist. **(B)** shows the model nullclines for the two examples and the corresponding mixed culture dynamics. **(C)** presents the density in % for both cells in mixed culture after around 10 *days*, as a function of temperature and considering two different co-inoculation conditions. The percentage of *Sk* exceeds that of *Sc* at cold temperatures. While the opposite behavior is found for *T* > 12°C. For *T* > 24°C *Sk* is excluded. **(D)** presents an illustrative example of sequential inoculation at 25°C showing how both species may coexist.

Doubling times decrease when the temperature increases for both species, being values for *Sc* always larger than for *Sk*. Doubling times for *Sc* and *Sk* differ significantly at low temperatures. At 8°C *Sc* requires 81.19 *h* to double population while *Sk* requires only 32.47 *h*. Interestingly, doubling times almost coincide for both species in the range [22 − 25]°C; being the values below 4 *h*. 10*x* times also decrease with the temperature for both cells; however, 10*x* times for *Sc* are shorter than those for *Sk* at higher temperatures. For example, at 25°C, *ts*_10*x,Sc*_ = 8.58 *h* while *ts*_10*x,Sk*_ = 11.14 *h*. Figure 3 shows the ratio between 2*x* and 10*x* times for both cells in single and mixed cultures. Results show that the ratio of doubling times is pretty similar in single and mixed culture; and the ratio>1 for all cases, indicating that *Sc* is slower in doubling its population than *Sk*. Despite this result at early times of the process, for temperatures above 16°C, *Sc* is faster than *Sk* in multiplying the initial population by 10. This difference is even higher in mixed cultures.

To explore this in more detail, we also computed the increase in the doubling and 10*x* times experienced by each species due to the presence of the other ($r\Delta t_{2x,i}=100\times \frac{tm_{2x,i}-ts_{2x,i}}{ts_{2x,i}}$ and $r\Delta t_{10x,i}=100\times \frac{tm_{10x,i}-ts_{10x,i}}{ts_{10x,i}}$). Figure 2G presents the results. The relative increase in doubling time is lower for *Sc* independently of the temperature. *Sk* increases the doubling time in around 1% due to the presence of *Sc* regardless of the temperature, while *Sc* doubling time increases between the 0.17 (at 8°C) and the 0.89% (at 25°C) due to the presence of *Sk*. When considering 10*x* times, the relative increase for *Sc* is lower than 1.92% while for *Sk* ranges from 1.43 and 5.65%.

### 3.4. Temperature Shapes Coexistence in Mixed Culture Fermentations

The inspection of the interspecific competition parameters reveals the competitive strength of *Sc* over *Sk* (*a*_*Sk,Sc*_) is independent of temperature. In contrast, the competitive strength of *Sk* over *Sc* (*a*_*Sc,Sk*_) increases quadratically with the temperature. Interestingly, and regardless the temperature, *a*_*Sc,Sk*_ ≤ 2.895 in the considered range of temperatures, which is around half the strength of the interaction of *Sc* over *Sk*. This has implications on the coexistence/exclusion of the species.

The geometric analysis of the nullclines of the best model allows characterizing the global behavior of the competing species, i.e., identifying the conditions leading to the exclusion of one species. The nullclines are the lines (for the LV model) or the curves (for the GA and SGA models) where $\frac{d_{x_{i}}}{dt}=0,i=1,2$, i.e., show the boundary between the increase and decrease in *x*_*i*_. We can obtain two nullclines, each for each species, as a function of *x*_*Sc*_. If the nullclines intersect each other, both species coexist, and the intersection corresponds to the equilibrium point. If the nullclines do not intersect with each other, one species excludes the other, and the relative position of the nullclines indicates which is the species being excluded. Remark that the result is independent of the initial inoculum in co-inoculation (i.e., with *x*_*i*,0_ > 0). In our particular case, because biological parameters depend on the temperature, we define two temperature-dependent nullclines as follows:

(15) $$\begin{array}{l} Sc\;nullcline\;:\;\frac{dx_{Sc}}{dt}=0;\\ x_{Sk}\left(T\right)=\frac{\gamma_{Sk}}{\left[a_{Sc,Sk}\left(T\right)\left(1-{\left(\frac{x_{Sc}}{K_{Sc}\left(T\right)}\right)}^{\theta_{Sc}}\right)-1\right]} \end{array}$$

(16) $$\begin{array}{l} Sk\;nullcline\;:\;\frac{dx_{Sk}}{dt}=0;\\ x_{Sk}\left(T\right)=K_{Sk}\left(T\right){\left[1-a_{Sk,Sc}\frac{x_{Sc}}{x_{Sc}+\gamma_{Sc}}\right]}^{\frac{1}{\theta_{Sk}}} \end{array}$$

Figure 3A presents the geometrical configuration of the nullclines as a function of the temperature. An inspection of the figure reveals that nullclines intersect each other in the range *T* ∈ [8, 24]°C, while for *T* > 24°C the *Sc nullcline* is above the *Sk nullcline* indicating that *Sc* excludes *Sk*. Figure 3B shows a couple of examples at *T* = 24.5°C and *T* = 16°C, respectively. In the first example, the *Sc nullcline* is above the *Sk nullcline*, indicating that *Sc* excludes *Sk*, as shown in the system dynamics. In the second example, both nullclines intersect each other, and both cells coexist.

To further illustrate the fact that coexistence or exclusion are independent of the initial relative amount of the competing species in co-inoculation, we present, in Figure 3C, the percentage of each cell for different temperatures after 10 *h*. We considered the following two inoculation: *Sc/Sk* 25/75 and 15/85. Results reveal that *Sc* never gets excluded, although its final amount depends on the temperature. The higher the temperature, the larger the final density of *Sc*. *Sk* dominates fermentation at cold temperatures. The model predicts that the most favorable case corresponds to the 15/85 fermentation at 8°C in which the final *Sk* density is 10 times higher than that for *Sc*. As predicted by the nullclines, for *T* > 24°C, *Sk* is excluded and it almost disappears after 10 *h*.

Cold temperatures as low as 8 − 12°C are rarely used in industrial settings. We therefore decided to explore the possibility of increasing the final amount of *Sk* at mild temperatures. We planned a sequential inoculation at 25°C in which *Sc* is inoculated into the system once *Sk* has already multiplied by 10 its initial population. Figure 3D presents the results, illustrating, how it is possible to overcome exclusion with sequential inoculation. The experimental results show how after 240*h* both cells coexist while in co-inoculation *Sk* had substantially decayed after 140*h*.

## 4. Discussion

Prior studies have noted the importance of the temperature in the dynamics of mixed cultures of *S. cerevisieae* and *non-cerevisiae* species (Arroyo-López et al., 2011; Alonso-del-Real et al., 2017). These studies analyzed the individual growth curves using single experiment data fitting to a Gompertz model (Arroyo-López et al., 2011; Alonso-del-Real et al., 2017). However, the approach is local in nature, i.e., derived models cannot be used to make predictions of systems behavior under different operating conditions nor to predict the ecology of mixed cultures.

The present study was designed to investigate the ecology of two *Saccharomyces* yeast species in mixed culture cold fermentations through a model-based approach. We used an iterative model building and selection procedure, based on multi-experiment data fitting, to find the model that offers the best compromise between complexity, goodness-of-fit and cross-validation.

The Gilpin-Ayala model clearly outperformed the classical Lotka-Volterra model. It should be noted that both models are fundamentally different. The LV model assumes that single growth, i.e., the intraspecific competence, depends linearly on the cellular density. In this case, the per-capita growth rate decreases linearly with the cellular density, and the maximum growth rate is achieved when the cellular density corresponds to half the carrying capacity *x*_*i*_ = *K*_*i*_/2. In the GA model the intraspecific competence depends non-linearly on the cellular density, thus the per-capita growth rate decreases non-linearly with the density and the maximum growth rate is achieved at $x_{i}={\left(\frac{1}{1+o_{is}}\right)}^{1/o_{is}}K_{i}$. In fact, as *o*_*is*_ → 0, the growth curve tends toward the Gompertz growth curve that achieves its maximum at *x*_*i*_ = *K*_*i*_/*e* (Zwietering et al., 1990).

Our results suggest that non-linear effects are particularly relevant at low densities (*o*_*i*_ < 1 for both strains) and that the Gompertz model might not be particularly well-suited to describe the dynamics of individual growth in mixed cultures. Also, the fact that *o*_*Sc*_ < *o*_*Sk*_ implies that regulation is faster for *Sc* than for *Sk*. This would be enough to compensate for the lag-phase for *T* >= 14°C for which *Sc* achieves the maximum growth rate earlier than *Sk*.

Both the specific growth rate (μ) and the carrying capacity (*K*) embed information about nutrient use and cellular decay. Thus the results in single growth suggest that both species differ in their abilities to exploit available nutrients and the magnitude of decay. Indeed these abilities are strongly affected by temperature.

In what regards to the specific growth rate dependence with the temperature results are in good agreement with previous works in the range below optimal growth temperature (Salvadó et al., 2011). We also obtained that the carrying capacity varies with the temperature. This fact is typically not considered in the food predictive microbiology literature, i.e., secondary models are generally used for μ (Ross and Dalgaard, 2003). Our secondary model selection procedure excluded this possibility; results revealed that the carrying capacity augmented with the temperature following an Arrhenius type dependency for both species. This finding is consistent with that of Zakhartsev et al. (2015) who also noted that the biomass yield for *S. cerevisiae* increases with the temperature below the optimal growth temperature. The authors related this behavior to higher cellular maintenance costs far from the optimal growth temperature. The fact that the growth rate for *Sk* is higher than for *Sc* at very low temperatures, might indicate that this effect is enhanced for *Sc* as compared to *Sk*.

In mixed cultures, both cells are affected by the presence of the other. However, the effects are uneven: the competitive strength of *Sc* over *Sk* is higher than the competitive strength of *Sk* over *Sc*. For *Sc*, the effect of the temperature is much higher than the effect of *Sk*: doubling and 10*x* times are only slightly affected (<2%) by the presence of *Sk* and the final yield of *Sc* is always higher than that of *Sk*. On the contrary, the presence of *Sc* exerts several effects on the dynamics of *Sk*: (1) the time required to multiply the initial population by ten increases up to a 6%; (2) the population experiences an overshoot and a decay whose intensity depends on the temperature; (3) *Sk* is excluded at higher temperatures (above 24°C) in co-inoculation independently of the initial inoculum.

In ecology, coexistence and exclusion relate to the concept of ecological niche. Ecological niche corresponds to the joint description of the environmental conditions that allow a species to satisfy its minimum survival requirements along with the effects of that species on these environmental conditions (Chase and Leibold, 2003).

In our particular case, niche partitionings can be due to the following initial environmental factors: temperature and available nutrients and to a whole variety of effects on the environment: production of ethanol or other toxic compounds, cell-to-cell contact or flocking effects, etc. Niche differences cause species to limit themselves (intraspecific competence) more than they limit competitors (interspecific competence) allowing for coexistence (Hector and Hooper, 2002; Levine and HilleRisLambers, 2009).

In what regards to temperature, our model (see Figure S5) predicts two different scenarios. At low temperatures ([8 − 10^*o*^*C*]), the effect of *Sc* over *Sk* is lower than the *Sk* intraspecific competence during the standard fermentation times (around 10 days). Thus, both cells can coexist. Interestingly, the final biomass yield is higher than that achieved in single cultures, as if both species experiment a mutual benefit in mixed cultures at low temperatures. At higher temperatures *T* > 24°C, the effect of *Sc* over *Sk* is higher than the *Sk* intraspecific competence for most of the process duration; thus *Sk* is excluded.

These results imply that temperature is a differential niche property for both cells. This accords with previous observations which concluded that a significant shift in the adaptation to growing at higher temperatures occurred in the *Saccharomyces* genus after the divergence of *S. cerevisiae* lineages from the *S. kudriavzevii* and *S. bayanus var. uvarum* lineages (Salvadó et al., 2011).

In what concerns the ability to exploit nutrients, our results show only a slight modification in the per-capita growth rate in single and mixed cultures, particularly at early times. This would indicate that both cells have a predefined nutrient consumption pattern and that this pattern would be a differential niche. This finding is consistent with earlier observations (Tronchoni et al., 2009; Henriques et al., 2018; Alonso-del-Real et al., 2019). In their work Tronchoni et al. (2009) showed that both species prefer glucose to fructose, while the transport of both hexoses increases with the temperature in the range [8 − 25]°C (Henriques et al., 2018). Besides, Alonso-del-Real et al. (2019) used transcriptomic analysis and HPLC determination of metabolites present in the must during the fermentation to identify the different pattern of nitrogen source preference between the species. Interestingly, the authors observed that nitrogen source and sugar consumption in mixed cultures showed a very similar profile to that exhibited by *S. cerevisiae* in single cultures.

The fact that for *Sk* the difference between intra- and interspecific competence becomes negative late in the process at lower temperatures, may originate in the limited tolerance of *Sk* to ethanol (Arroyo-López et al., 2010) or the possible production of other kind of toxic compound such as the already described small peptides derived from the enzyme glyceraldehyde-3-phosphate dehydrogenase (Albergaria et al., 2010; Branco et al., 2014). The ability of *Sc* to divert the metabolism to the production of ethanol and toxic compounds would be then a niche characteristic of the species.

Niche partitioning would also explain why it is possible to overcome exclusion by sequential inoculation. 24*h* after *Sk* inoculation, more than half the glucose and the fructose have already been consumed (Henriques et al., 2018). Possibly also assimilable nitrogen sources have been significantly reduced. When *Sc* enters the process the available nutrients, particularly the nitrogen sources, affect the specific growth rate and the carrying capacity. Also the production of ethanol is limited by the remaining amount of hexoses. The experimental results showed how after 240*h* both cells coexist while in co-inoculation *Sk* had substantially decayed after 140*h*. Further investigation should focus on developing mechanistic models accounting for niche partitioning—temperature, differential nutrient consumption, and ethanol production—so as to enable the automatic design of sequential mixed cultures.

## 5. Conclusions

The main goal of the current work was to explore the role of the temperature in the dynamics of mixed fermentations through an ecological model-based approach.

We considered the particular case of mixed culture fermentations involving *S. cerevisiae* and *S. kudriavzevii* strains. The best model corresponded to a Gilpin-Ayala model with saturable competition; different secondary models were obtained for the different biological parameters.

The analysis of the model revealed that *Sc* excludes *Sk* at temperatures higher than 24°C in co-inoculation. We also obtained major differences in growth properties in single and mixed cultures for both species concluding that temperature, nutrient consumption patterns and tolerance to toxic compounds are differential niches for both species which affect per-capita growth rate and decay.

The principal implication of this research is that sequential inoculation is the most promising alternative for novel fermentations at mild temperatures. Although the study focuses on a particular strain combination, the findings may well have a bearing on other cases.

The ecological models considered in this work are particularly well-suited to explore coexistence and exclusion conditions. Further research is needed to formulate mechanistic models suited for optimal model-based design of novel mixed fermentations.

## Data Availability Statement

The datasets presented in this study can be found in online repositories. The names of the repository/repositories and accession number(s) can be found below: https://sites.google.com/site/amigo2toolbox/examples.

## Author Contributions

EB-C and AQ designed the work. EB-C formulated the models and performed the numerical analyses. AQ and JA-R conceived and designed the experiments. JA-R performed the experiments. All authors analyzed the results and drafted the manuscript.

## Conflict of Interest

The authors declare that the research was conducted in the absence of any commercial or financial relationships that could be construed as a potential conflict of interest.
