# Supplementary material for: Temperature Shapes Ecological Dynamics in Mixed Culture Fermentations Driven by Two Species of the Saccharomyces Genus
Source: Front Bioeng Biotechnol. 2020 Aug 21;8:915. doi: 10.3389/fbioe.2020.00915 (PMC7472092; doi:10.3389/fbioe.2020.00915)
Supplement: Supplementary file 1 [file Image_1.pdf]

Supplementary file

Temperature shapes ecological dynamics in mixed culture fermentations driven by two species of the *Saccharomyces* genus

E. Balsa-Canto, J. Alonso-del-Real and A. Querol

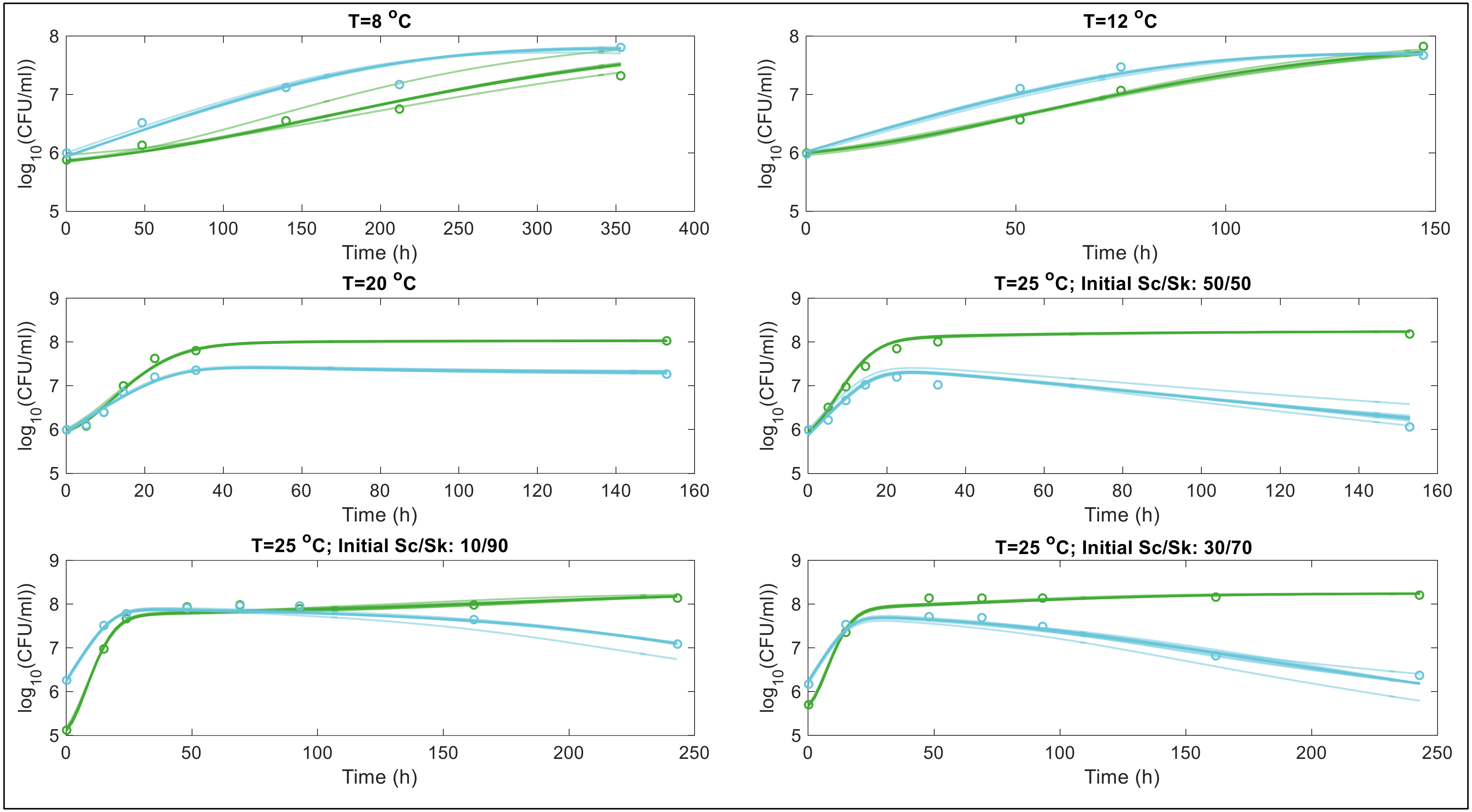

Figure S1. Results of the cross-validation for mixed culture experiments. Green data and curves correspond to *Sc*, while those in blue correspond to *Sk*. Dots represent experimental data; continuous lines represent model predictions for the different cross-validation tests.

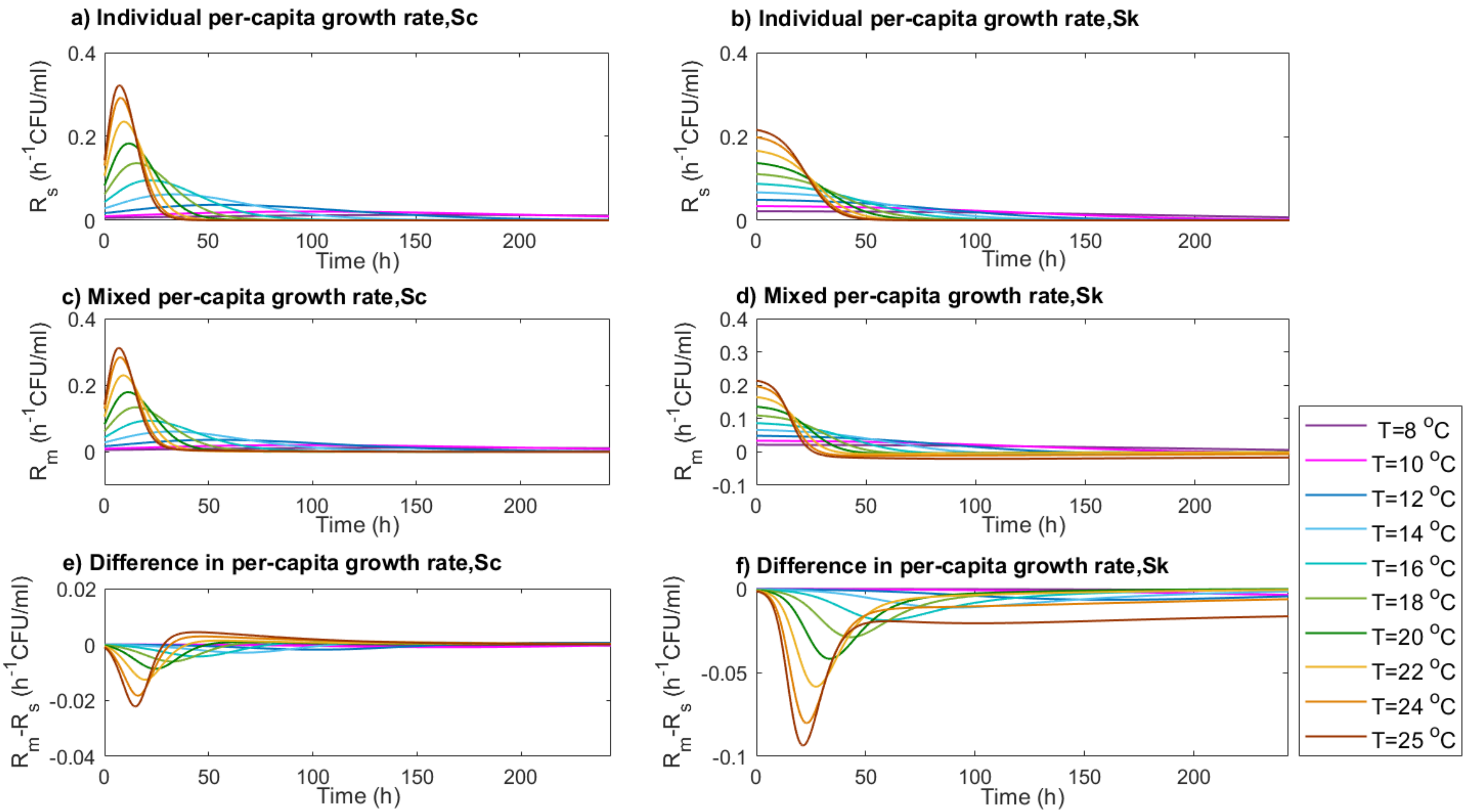

Figure S2. Per-capita growth rate as a function of time and temperature. Figures a) and b) present the per-capita growth rate in individual cultures. Figures show that the maximum per-capita growth rate is larger for *Sc* than for Sk. In the case of *Sk*, the maximum per-capita growth rate is achieved at the beginning of the process and later decays non-linearly towards 0. For *Sc*, the maximum per-capita growth rate is achieved later due to the effects of the lag-phase. Figures c) and d) present the per-capita growth rate in mixed co-inoculated fermentations (50/50). Remark that for the case of *Sk*, at higher temperatures, the per-capita growth rate becomes negative, indicating a fast decay of the *Sk* population. Figures e) and f) present the difference between the per-capita growth rate in mixed and individual cultures. For the case of *Sk*, the per-capita growth rate is always lower in mixed culture, while this is not the case for *Sc*.

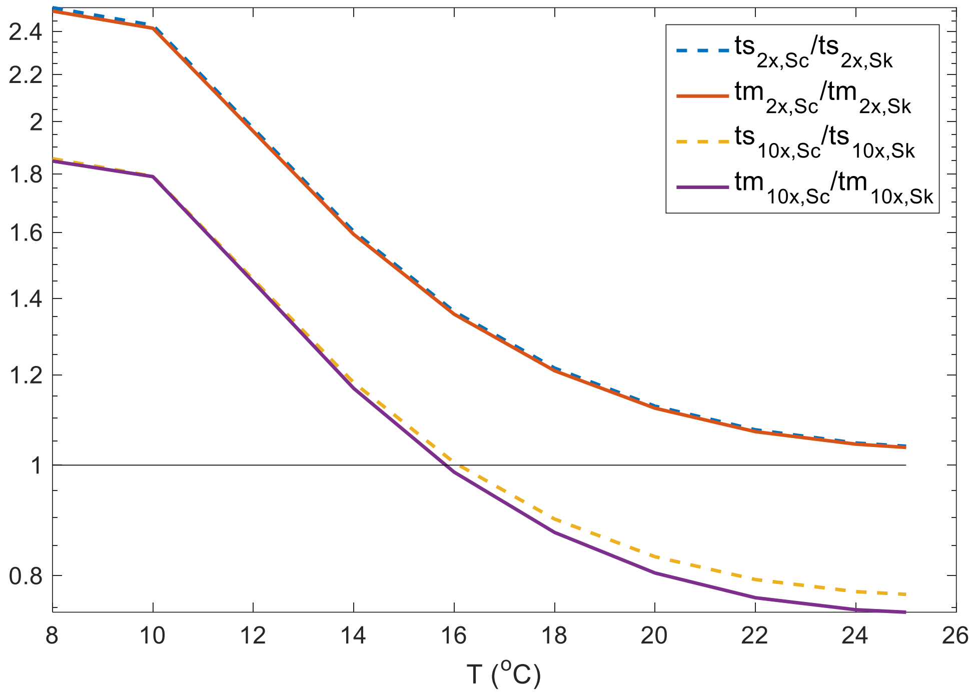

Figure S3. Ratio between 2x and 10 times in single and mixed cultures. Results show that the ratio of doubling times is pretty similar in single and mixed culture; and the ratio>1 for all cases, indicating that Sc is slower in doubling its population. Curves corresponding to the ratios between x10 times show that for temperatures above 16 ^{c}C, *Sc* multiplies by 10 the initial population faster than *Sk*. This difference is even higher in mixed cultures.

The initial inoculum corresponds to X_{i,0}=10^6 CFU/ml for both species.

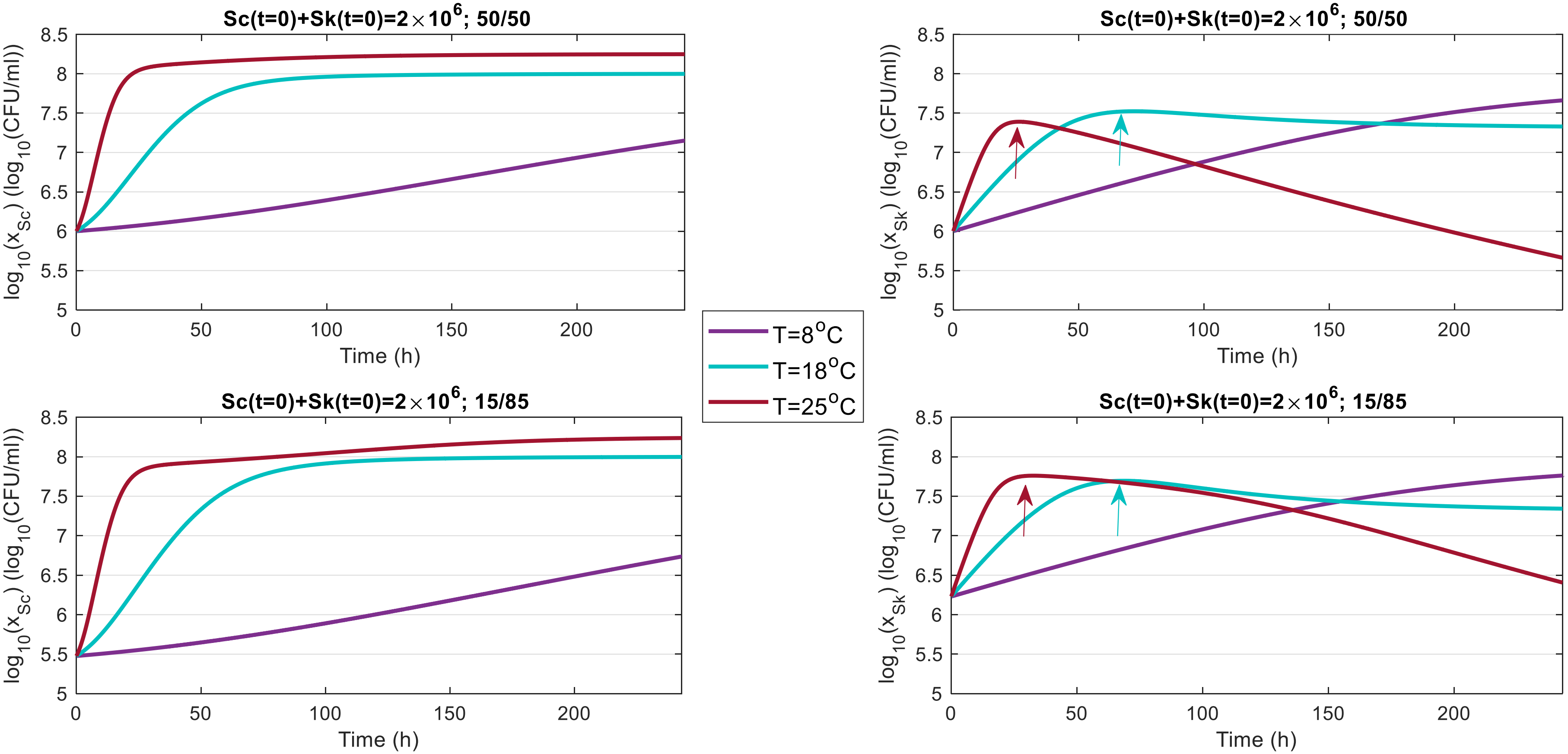

Figure S4. Simulation of 10 days process under different temperatures and initial co-inoculation conditions. The model predicts that *Sk* experiences an overshoot (see arrows) and a collapse at mild temperatures. The intensity of the collapse increases with the temperature and *Sk* is excluded at the highest temperature independently of the initial inoculation.

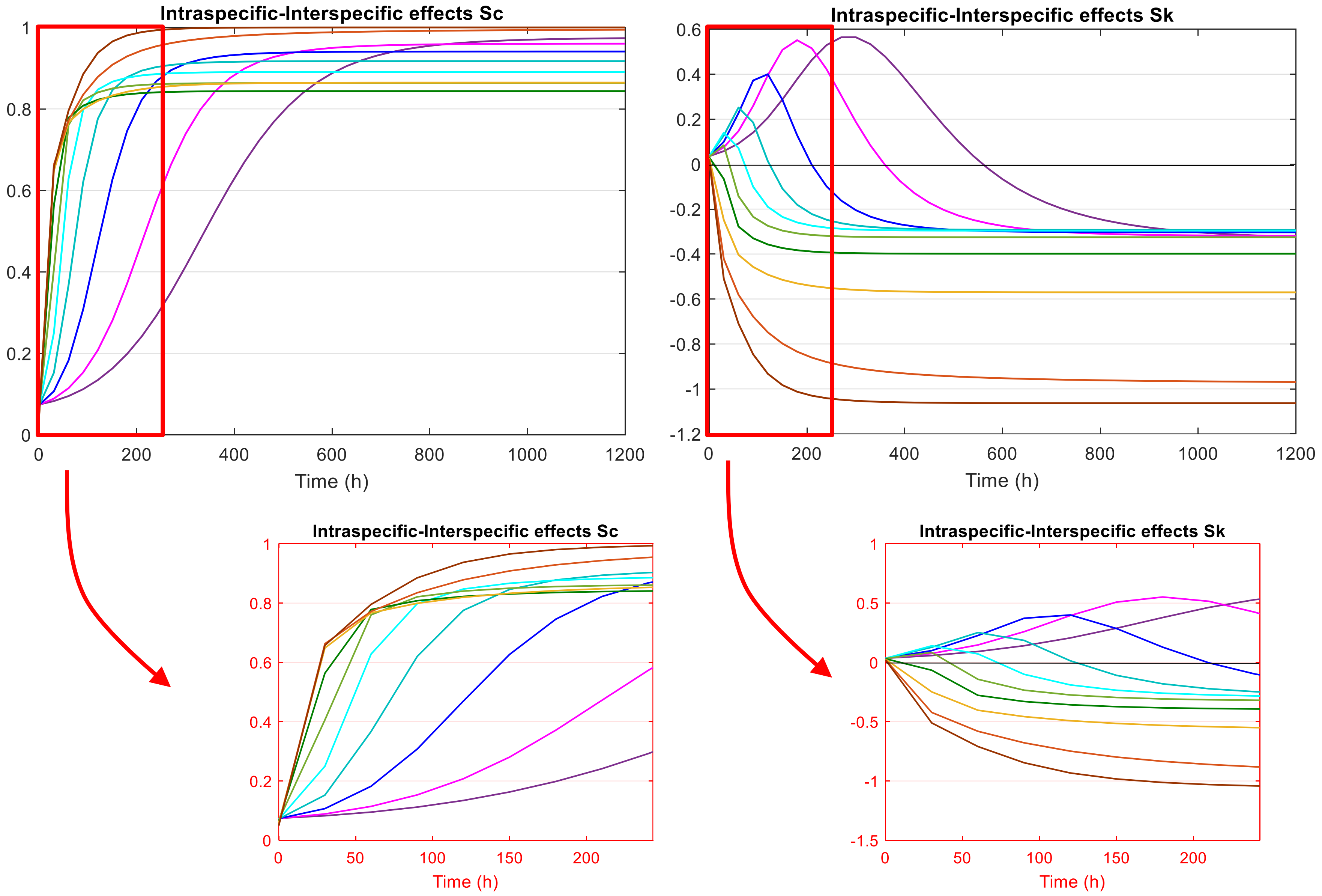

Figure S5. Intraspecific – Interspectific effects as a function of the temperature. Niche differences cause species to limit themselves (intraspecific competence) more than they limit competitors (interspecific competence). For the case of *Sc*, intraspecific competence is higher than the interspecific competence. On the contrary, the result strongly depends on the temperature for *Sk*.

The initial inoculum corresponds to X_{i,0}=10^6 CFU/ml for both species.
